# Barriers to and enablers of the early diagnosis of breast cancer among women from ethnic minority backgrounds in the UK: protocol for a qualitative evidence synthesis

**DOI:** 10.1136/bmjopen-2024-092480

**Published:** 2024-11-07

**Authors:** Priya Sajjad, S Shepperd, Shoba Dawson, Bep Dhaliwal, Nia Roberts, Toral Gathani

**Affiliations:** 1 Cancer Epidemiology Unit, Nuffield Department of Population Health, University of Oxford, Oxford, UK; 2 Nuffield Department of Population Health, University of Oxford, Oxford, UK; 3 Sheffield Centre for Health and Related Research, University of Sheffield, Sheffield, UK; 4 Patient, Oxford, UK; 5 Bodleian Health Care Libraries, University of Oxford, UK, Oxford, UK; 6 Cancer Epidemiology Unit, Nuffield Department of Population Health, University of Oxford, OXFORD, UK; 7 Oxford University Hospitals NHS Foundation Trust, Oxford, UK

**Keywords:** Breast tumours, Health Equity, QUALITATIVE RESEARCH

## Abstract

**Abstract:**

**Introduction:**

Breast cancer is the most commonly diagnosed cancer in women of all ethnic groups in the UK. The largest single ethnic groups in the UK are white, Indian, Pakistani, black African and black Caribbean. Previous studies have shown that women from ethnic minority groups are more likely to be diagnosed with more advanced disease at presentation compared with women from white backgrounds which is associated with poorer outcomes. Understanding the factors that prevent or enable women from ethnic minority backgrounds to have an early diagnosis of breast cancer is essential to inform the development of interventions or policies that seek to promote early diagnosis of breast cancer in these groups. This qualitative evidence synthesis will identify and synthesise what is known about the topic.

**Methods and analysis:**

The proposed review will synthesise studies that report on the experiences of women in the UK from the ethnic groups of interest in the early diagnosis of breast cancer. A search strategy was developed by two researchers and an information specialist. The Medline (OvidSP), Embase (OvidSP), CINAHL (EBSCOHost), PsycINFO (OvidSP) and Cochrane Library (via Wiley) databases will be searched for published articles. Relevant policy documents and reports will be searched for by browsing cancer-related organisational websites and using Google Advanced Search, and dissertations and theses using ProQuest. Studies will be included if they use qualitative methodologies and are about the early diagnosis of breast cancer in UK women aged 18 years and over from the ethnic minority groups of interest. Studies that use quantitative methodologies or collect data using qualitative methods but analysed quantitatively (eg, open-ended survey questions where free-text responses are analysed using descriptive statistics) will be excluded. To maximise the inclusion of suitable studies, the search will not be limited by language and start from database inception. Data will be managed using Covidence and managed for analysis using NVivo. An assessment of the methodological limitations of each study will be performed using the Critical Skills Appraisal Programme tool, and the PRO EDI framework will be used to assess equality, diversity and inclusion in the synthesis. The data will be analysed thematically based on, but not limited to, the health belief model, using a best-fit framework. The confidence in the final synthesised findings will be assessed using the Grading of Recommendations Assessment, Development, and Evaluation, Confidence in the Evidence for Reviews of Qualitative research tool.

**Ethics and dissemination:**

Ethical approval is not required as this is a systematic review of published or publicly available qualitative findings. Results will be published in a peer-reviewed scientific journal, publicised at relevant conferences and on social media. The results will provide comprehensive information on the barriers to early diagnosis of breast cancer in ethnic minority groups, which will potentially inform breast cancer care policies to improve access and delivery of health services and influence the design of future interventional and qualitative studies.

**PROSPERO registration number:**

CRD42024579776.

**STRENGTHS AND LIMITATIONS OF THIS STUDY**

- This qualitative evidence synthesis will incorporate published scientific literature, policy documents and research theses to maximise the completeness and diversity of data.
- The protocol has been developed by an interdisciplinary group of researchers.
- The review will contextualise the findings in relation to routes to diagnosis for breast cancer which will vary by ethnicity.
- The review will be limited by the quality of the studies available for data synthesis.

## Introduction

 Breast cancer is the most commonly diagnosed cancer in women in all ethnic groups in the UK.^1^ Around 50, 000 women are diagnosed with breast cancer each year.^2^ Breast cancer survival has improved in more developed countries by about 40% over the last four decades, and this improvement is largely attributable to strategies to promote earlier detection and early diagnosis and better access to effective treatments.^3^

Although the terms early detection and early diagnosis are often used interchangeably, they do have distinct meanings.^4^ Early detection refers to the diagnosis of disease through population-based screening programmes in individuals without symptoms. Early diagnosis refers to the prompt diagnosis of patients with symptoms and is facilitated through smooth referral pathways from primary to secondary care.

In the UK, women can be referred for assessment of breast symptoms, such as a lump, at any age. Additionally, women aged 50–70 years are invited for mammographic screening every 3 years. Overall, 6 out of 10 breast cancer diagnoses arise as a result of symptoms, and 3 out of 10 breast cancer diagnoses are a result of screening. However, among women of screening age (50–70 years), 60% of breast cancer diagnoses are as a result of screening.^5^

The largest single ethnic minority groups in the UK are white, Indian, Pakistani, black African and black Caribbean. Census data show that the ethnic minority populations are, on average, significantly younger, compared with the majority white population.^6^ These differences will influence the route to diagnosis for breast cancer in different ethnic groups, as women from ethnic minority backgrounds are less likely to be of screening age and more likely to present with symptoms.

Studies have also shown that women from ethnic minority backgrounds are more likely to be diagnosed with more advanced stages of disease at presentation which is associated with poorer outcomes.^7 8^ This may be due, in part, to delays seeking help for breast symptoms, or lower attendance at screening among those women who are eligible.

Existing reviews are limited by either solely focusing on screening attendance^9^ or on particular ethnic groups.^10 11^ Furthermore, since these reviews were published, there has been an increasing interest in addressing cancer inequalities in underserved populations. As such, there is an expanding evidence base of peer-reviewed scientific literature and reports from the third sector in this area and a comprehensive review is therefore timely.

This review aims to fill a knowledge and evidence gap by focusing on the reported experiences of women from the largest single ethnic groups in the UK of the early diagnosis of breast cancer. This review will summarise the barriers and facilitators to the early diagnosis of breast cancer in different ethnic groups and aims to directly inform breast cancer care policies to improve access to and delivery of services, and the design of future interventional and other primary studies.

## Methods and analysis

### Registration

This protocol is registered on PROSPERO (CRD42024579776).

### Criteria for considering studies for the qualitative evidence synthesis

Studies will be included if they:

- Use qualitative methodologies.
- Examine the phenomenon of interest in UK women aged 18 years and over.
- Include the ethnic minority groups of interest.

The Sample, Phenomenon of Interest, Design, Evaluation, Research type search tool^12^ was used to define the search terms and is described in table 1.

**Table 1:** The SPIDER search tool

Sample (S)	Women aged ≥18 years of age and belonging to these ethnic minority groups: Indian, Pakistani, Black African and Black Caribbean and resident in the UK.
Phenomenon of Interest (Pi)	Early breast cancer diagnosis, focusing on why women from ethnic minority groups delay seeking advice for breast symptoms.
Design (D)	Qualitative studies using data collection methods such as interviews and focus groups, ethnography and observations, etc.
Evaluation (E)	The synthesis will evaluate barriers and facilitators to early breast cancer diagnosis which relates primarily to symptomatic presentation.
Research type (R)	The search will focus on qualitative research and mixed methods research but only where the qualitative element is clearly defined and reported.

Studies will be excluded if they:

- Use quantitative methodologies.
- Are not about early breast cancer diagnosis or collect data using qualitative methods but analysed quantitatively (eg, open-ended survey questions where free-text responses are analysed using descriptive statistics).

### Search strategy for identification of studies

A search strategy was developed by two reviewers (TG/PS) with input from an information specialist (NR). The search strategy is described for all potential information sources for relevant articles including the published scientific literature, research theses and policy documents and reports.

### Information sources

To identify articles from the published scientific literature, the electronic databases of Medline (OvidSP), Embase (OvidSP), CINAHL (EBSCOHost), PsycINFO (OvidSP) and Cochrane Library (via Wiley) will be searched. The MEDLINE search strategy (online supplemental appendix 1) will be adapted for the other databases with the aid of Polyglot SR.^13^ To identify dissertations and theses, a topic search will be conducted in ProQuest Dissertations & Theses (Global). The dissertations and theses will be limited to those published in England, Scotland or Wales.

To search for relevant policy documents and reports available online, we will use Google Advanced Search, using a combination of terms including “breast cancer” and “early diagnosis” and “ethnicity”, with a filter applied to the UK region (online supplemental appendix 1). The first 10 pages of search results (representing 100 hits) will be reviewed by title and description to identify the most relevant entries while maintaining a manageable number to the screen. We will also conduct a review of targeted websites (online supplemental appendix 1), first by identifying and selecting relevant health organisations and charities within the UK that publish reports on breast cancer, and second by conducting a detailed review of each website.

The reference lists of included studies will be reviewed to identify further relevant studies, and forward citation searching will be conducted. To maximise the inclusion of suitable studies, the search will not be limited by language or time frame. A PRISMA (Preferred Reporting Items for Systematic Reviews and Meta-Analyses) flow chart template is provided in online supplemental appendix 2.

### Study selection

Search results from the databases will be imported into the Covidence platform^14^ and after deduplication, titles, abstracts and full-text articles will be screened against eligibility criteria for inclusion. One researcher will screen the titles and abstracts against the inclusion and exclusion criteria (PS), and a random 20% sample of these will be checked by a second researcher (TG). If there is a high level of discrepancy, titles and abstracts will be reviewed independently by a further two reviewers (SS/SD). Two review authors will then independently assess the full text of the studies assessed as potentially eligible (TG/PS). Disagreements will be resolved through discussion or, if necessary, by involving a third review author (SS).

### Quality assessment

The methodological limitations of each study will be assessed using the Critical Skills Appraisal Programme qualitative checklist tool by two reviewers working independently (PS/TG).^15^ A third member of the research team will be consulted in case of disagreements (SS).

### Data extraction and analysis

To manage and extract findings from the included articles, we will use Covidence to extract data from each study using a pro-forma template. We will use the PRO-EDI (Promoting Equality, Diversity, and Inclusion in Evidence Synthesis) framework to assist with the identification and classification of equity-relevant data (table 2).^16^ Examples of data to be extracted from each study include publication details (author, year of publication and country of publication), sampling method used, the study design, the analytical approach and the main findings. The proposed data extraction will be piloted in a small number of studies and discussed among the team to ensure appropriate and relevant information is captured.

**Table 2:** The PRO EDI (Promoting Equality, Diversity, and Inclusion in Evidence Synthesis) participant characteristics of interest

Characteristics	The people we would expect to see	The people who took part
Age		
Ethnicity		
Socioeconomic status		
Level of education		
Disability		
Location		
Other		

Using a best-fit a priori framework as our analytical approach, the findings for each study will be grouped (PS/SS) using the key concepts of, but not be limited to, the health belief model (HBM) to organise the findings from each study by relevant themes.^17 18^ The HBM is used extensively across countries and different populations, to explain barriers and enablers to health behaviours. We will generate a list of codes and then develop themes using NVivo (V.14)^19^ to aid comparison among studies. The approach will be flexible, and additional themes will be added as they are identified from the data. The findings will be reported in a tabular format with a narrative synthesis. We will report the findings of the qualitative synthesis in accordance with the ENTREQ (Enhancing Transparency in the Reporting the Synthesis of Qualitative Research)

checklist.^20^

### Assessment of confidence in synthesised findings

The Grading of Recommendations Assessment, Development and Evaluation, Confidence in the Evidence for Reviews of Qualitative research will be applied to judge confidence in the synthesised findings.^21^ The criteria will be applied to each study finding, assessing four key components including methodological limitations, relevance, coherence and adequacy of data. Confidence levels will be categorised as high, moderate, low or very low with a final assessment based on consensus among the research team. The assessment of the confidence of the findings will be reported in a table.

### Patient and public involvement

This qualitative evidence synthesis is part of a larger ethnicity and breast cancer research project which is supported by an ethnically diverse patient and public involvement (PPI) panel. Further details are available at https://www.ceu.ox.ac.uk/research/ethnicity-and-breast-cancer.

A patient contributor (BD) has read the manuscript and provided feedback on the protocol. The findings of the review will be shared with the study PPI panel to see to what extent the findings resonate with their own experiences and identify any key aspects that are missing from the literature.

## Ethics and dissemination

Ethical approval is not required as only published or publicly available qualitative findings will be included. The findings of the qualitative evidence synthesis will be disseminated via peer-reviewed scientific publication and presentations at relevant conferences. In addition, lay summaries and infographics will be cocreated with the PPI groups to disseminate findings through relevant community networks and leveraging the communication channels of the PPI panel, using traditional approaches and social media.

### Review author reflexivity

The review team is ethnically diverse and consists of researchers skilled in qualitative, and mixed methods/applied research and evidence synthesis. One member of the team is a consultant breast surgeon and well versed in the processes that lead patients to be diagnosed with breast cancer in the UK. The collective research experiences of the team may influence the choice of review methods and data interpretation. Consequently, each team member will be aware of how their personal perspectives and backgrounds might influence the review process. Decisions will be made through group discussions to ensure that various viewpoints from the review authors are considered.

## supplementary material

10.1136/bmjopen-2024-092480 online supplemental file 1

10.1136/bmjopen-2024-092480 online supplemental file 2
