# Supplementary material for: Barriers to and enablers of the early diagnosis of breast cancer among women from ethnic minority backgrounds in the UK: protocol for a qualitative evidence synthesis
Source: BMJ Open. 2024 Nov 7;14(11):e092480. doi: 10.1136/bmjopen-2024-092480 (PMC11551988; doi:10.1136/bmjopen-2024-092480)
Supplement: online supplemental file 1 [file bmjopen-14-11-s001.pdf]

Appendix 1: The search strategies

The Medline Search Strategy adopted for the other databases with the aid of Polygot SR.

("Breast Neoplasms"[MeSH Terms] OR ("breast cancer*"[Text Word] OR "breast neoplasm*"[Text Word] OR "breast tumour*"[Text Word] OR "breast tumor*"[Text Word])) AND ("Symptomatic"[Title/Abstract] OR ("Early Diagnosis"[MeSH Terms] OR "Early Diagnosis"[Text Word] OR "Early Detection"[All Fields] OR "early diagnoses*"[Text Word] OR "breast neoplasms/diagnosis"[MeSH Terms] OR "asymptomatic"[Text Word] OR "Asymptomatic Diseases"[MeSH Terms:noexp] OR "Mammography"[MeSH Terms:noexp] OR "Mass Screening"[MeSH Terms:noexp] OR ("mammogra*"[Title/Abstract] OR "screening*"[Title/Abstract]))) AND ("ethnic"[Text Word] OR "Ethnicity"[Text Word] OR "Ethnicities"[Text Word] OR "background*"[Text Word] OR "race*"[Text Word] OR "Racial"[Text Word] OR "Ethnicity"[MeSH Terms] OR ("black african*"[Text Word] OR "black caribbean*"[Text Word] OR "Pakistan"[Text Word] OR "Pakistanis"[Text Word] OR "British Pakistanis"[Text Word] OR "indian*"[Text Word] OR "british indian*"[Text Word]) OR "Caribbean People"[MeSH Terms] OR ("south asian*"[Text Word] OR "british south asian*"[Text Word] OR "british caribbean*"[Text Word] OR "british african*"[Text Word]) OR "Black People"[MeSH Terms] OR "Minority Groups"[MeSH Terms] OR "Ethnic and Racial Minorities"[MeSH Terms] OR ("Minority"[Text Word] OR "Minorities"[Text Word] OR "minority group*"[Text Word] OR "marginalise*"[Text Word] OR "marginalised group*"[Text Word] OR "discriminated group*"[Text Word] OR "discriminate*"[Text Word]) OR "Health Disparate Minority and Vulnerable Populations"[MeSH Terms]) AND ("barrier*"[Text Word] OR "impede"[Text Word] OR "impediment*"[Text Word] OR "facilitat*"[Text Word] OR "challenge*"[Text Word] OR "driver*"[Text Word] OR "limitation*"[Text Word] OR "hinder*"[Text Word] OR "obstacle*"[Text Word] OR "hurdle*"[Text Word] OR "opportuni*"[Text Word] OR "enabl*"[Text Word] OR "adapt*"[Text Word] OR ("barrier*"[Text Word] OR "inhibitor*"[Text Word] OR "challenge*"[Text Word] OR "complication*"[Text Word] OR "obstacle*"[Text Word] OR ("facilitator*"[Text Word] OR "promoter*"[Text Word] OR "enabler*"[Text Word]))) AND ("United Kingdom"[MeSH Terms] OR ("United Kingdom"[Text Word] OR "UK"[Text Word] OR "NHS"[Text Word] OR "National Health Service"[Text Word] OR "england"[Text Word] OR "scotland"[Text Word] OR "wales"[Text Word] OR "London"[Text Word] OR "Manchester"[Text Word] OR "Birmingham"[Text Word] OR "CARDIFF"[Text Word] OR "EDINBURGH"[Text Word] OR "northern ireland"[Text Word] OR "BELFAST"[Text Word]) OR ("United Kingdom"[Affiliation] OR "UK"[Affiliation] OR "NHS"[Affiliation] OR "england"[Affiliation] OR "scotland"[Affiliation] OR "wales"[Affiliation] OR "northern ireland"[Affiliation]))

Google Advanced Search strategy

Search number	Search terms
1	breast cancer AND early diagnosis AND ethnicity
2	breast cancer AND ethnicity AND early diagnosis
3	breast cancer AND ethnicity OR ethnic minorities
4	breast cancer AND Pakistanis AND early diagnosis
5	breast cancer AND Indians AND early diagnosis
6	breast cancer AND Black Africans AND early diagnosis
7	breast cancer AND Black Caribbeans AND early diagnosis

Relevant websites identified to review

	Organisation	Link
1	Breast Cancer UK	https://www.breastcanceruk.org.uk/
2	Prevent Breast Cancer	https://preventbreastcancer.org.uk/
3	Cancer Research UK	https://www.cancerresearchuk.org/
4	Breast Cancer Now	https://breastcancernow.org/
5	Macmillan Cancer Support	https://www.macmillan.org.uk/
6	Breast Cancer Prevention	https://breastcancerprevention.org.uk/
7	CoppaFeel!	https://coppafeel.org/
8	NHS	https://www.nhs.uk/
9	Breast Cancer Support	https://breastcancersupport.org.uk/
10	Against Breast Cancer	https://www.againstbreastcancer.org.uk/
11	Make 2nds Count	https://make2ndscount.co.uk/
12	Future Dreams	https://futuredreams.org.uk/
13	The Pink Ribbon Foundation	https://www.pinkribbonfoundation.org.uk/
14	Breast Cancer Hope Foundation	https://www.breastcancerhope.org.uk/
