# Supplementary figures and images for: Barriers to and enablers of the early diagnosis of breast cancer among women from ethnic minority backgrounds in the UK: protocol for a qualitative evidence synthesis
Source: BMJ Open. 2024 Nov 7;14(11):e092480. doi: 10.1136/bmjopen-2024-092480 (PMC11551988; doi:10.1136/bmjopen-2024-092480)

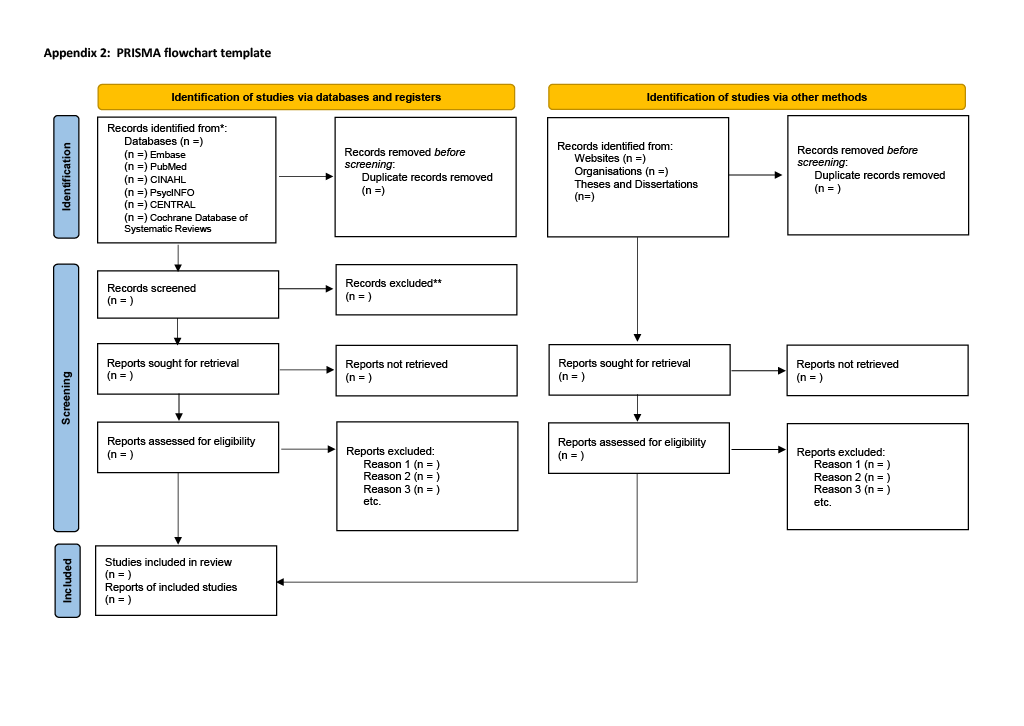

Supplement: online supplemental file 2 [file bmjopen-14-11-s002.png]
