# Supplementary material for: Barriers to and enablers of the early diagnosis of breast cancer among women from ethnic minority backgrounds in the UK: protocol for a qualitative evidence synthesis
Source: BMJ Open. 2024 Nov 7;14(11):e092480. doi: 10.1136/bmjopen-2024-092480 (PMC11551988; doi:10.1136/bmjopen-2024-092480)
Supplement: Supplementary file 3 [file bmjopen-2024-092480.reviewer_comments.pdf]

PEER REVIEW HISTORY

BMJ Open publishes all reviews undertaken for accepted manuscripts. Reviewers are asked to complete a checklist review form and are provided with free text boxes to elaborate on their assessment. These free text comments are reproduced below.

ARTICLE DETAILS

Title (Provisional)

Barriers to and enablers of the early diagnosis of breast cancer among women from ethnic minority backgrounds in the UK: protocol for a qualitative evidence synthesis

Authors

Sajjad, Priya; Shepperd, S; Dawson, Shoba; Dhaliwal, Bep; Roberts, Nia; Gathani, Toral

---

VERSION 1 - REVIEW

**Reviewer**	**1**
**Name**	**Hossain, Syeda Zakia**
**Affiliation**	**The University of Sydney, Health Sciences**
**Date**	**12-Sep-2024**
**COI**	**Not applicable.**

This is an important area for a systematic review of literature. The protocol developed followed the standard format and the presentation is well structured.

Researchers have discussed the ways to extract the literature relevant to the topic and how to review them for their eligibility. It is important to present the Prisma flow diagram for the study selection process.

Also eligibility of literature selection could be presented in a table for showing inclusion and exclusion criteria of the study/articles.

With regards to assessment of confidence in synthesised findings - it would be appropriate to present the results in a table form for comparative purpose and better understanding. Consider presenting the synthesised results in a table form and then analysed them.

Reviewer **2**

**Name**	**Davies, Elizabeth**
**Affiliation**	**King's College London, Health and Social Care Research**
**Date**	**21-Sep-2024**
**COI**	**I have no competing research interests**

Thank you for this very clear and well-conceived study protocol which should bring together, develop and add important information to the literature and help improve the earlier detection and diagnosis of breast cancer in women from the major UK ethnic groups. My only query which you might consider adding to the manuscript is to say why you decided not to include and compare quantitative survey data in this part of the study. While I agree that the qualitative data will provide detailed, nuanced, and incredibly useful insights, I just wonder whether the qualitative findings might also explain some of the survey findings on for example screening uptake. I realise that this may be covered in other parts of your study or be outside the scope but in general we often see mixed methods studies as giving us a greater overall understanding of any issue we are trying to solve or improve.

VERSION 1 - AUTHOR RESPONSE

Reviewer: 1
Dr. Syeda Zakia Hossain, The University of Sydney, University of Sydney
Comments to the Author:
This is an important area for a systematic review of literature. The protocol developed followed the standard format and the presentation is well structured.

Researchers have discussed the ways to extract the literature relevant to the topic and how to review them for their eligibility. It is important to present the Prisma flow diagram for the study selection process.
Authors' response: The Prisma flow diagram template has been provided as Appendix 2 and a sentence added to the manuscript Page 5 Line 146.

Also eligibility of literature selection could be presented in a table for showing inclusion and exclusion criteria of the study/articles.
Authors' response: We thank the reviewer for this suggestion but feel as the number of inclusion and exclusion criteria are small, a table is not needed in addition to the text description but have added bullet points for clarification Page 4 Lines 114-126.

With regards to assessment of confidence in synthesised findings - it would be appropriate to present the results in a table form for comparative purpose and better understanding. Consider presenting the synthesised results in a table form and then analysed them.
Authors' response: We have modified the sentence for clarity on Page 4 lines 189-190 to say "The assessment of the confidence of the findings will be reported in a table".

Reviewer: 2
Dr. Elizabeth Davies, King's College London, Public Health England
Comments to the Author:
Thank you for this very clear and well-conceived study protocol which should bring together,

develop and add important information to the literature and help improve the earlier detection and diagnosis of breast cancer in women from the major UK ethnic groups.

My only query which you might consider adding to the manuscript is to say why you decided not to include and compare quantitative survey data in this part of the study. While I agree that the qualitative data will provide detailed, nuanced, and incredibly useful insights, I just wonder whether the qualitative findings might also explain some of the survey findings on for example screening uptake. I realise that this may be covered in other parts of your study or be outside the scope but in general we often see mixed methods studies as giving us a greater overall understanding of any issue we are trying to solve or improve.
Authors' response: We thank the reviewer for this comment. Quantitative survey data is out of scope for this qualitative evidence synthesis that is focussed on the early diagnosis, rather than the early detection, of breast cancer in particular ethnic groups.

*** ***
COI statements:
Reviewer: 1
Competing interests: Not applicable.
Reviewer: 2
Competing interests: I have no competing research interests.
